# Changes in H^+^, K^+^, and Ca^2+^ Concentrations, as Observed in Seizures, Induce Action Potential Signaling in Cortical Neurons by a Mechanism That Depends Partially on Acid-Sensing Ion Channels

**DOI:** 10.3389/fncel.2021.732869

**Published:** 2021-10-15

**Authors:** Omar Alijevic, Zhong Peng, Stephan Kellenberger

**Affiliations:** Department of Biomedical Sciences, University of Lausanne, Lausanne, Switzerland

**Keywords:** ASIC, acidification, action potential, neuronal signaling, modification of gating by ions, calcium

## Abstract

Acid-sensing ion channels (ASICs) are activated by extracellular acidification. Because ASIC currents are transient, these channels appear to be ideal sensors for detecting the onset of rapid pH changes. ASICs are involved in neuronal death after ischemic stroke, and in the sensation of inflammatory pain. Ischemia and inflammation are associated with a slowly developing, long-lasting acidification. Recent studies indicate however that ASICs are unable to induce an electrical signaling activity under standard experimental conditions if pH changes are slow. In situations associated with slow and sustained pH drops such as high neuronal signaling activity and ischemia, the extracellular K^+^ concentration increases, and the Ca^2+^ concentration decreases. We hypothesized that the concomitant changes in H^+^, K^+^, and Ca^2+^ concentrations may allow a long-lasting ASIC-dependent induction of action potential (AP) signaling. We show that for acidification from pH7.4 to pH7.0 or 6.8 on cultured cortical neurons, the number of action potentials and the firing time increased strongly if the acidification was accompanied by a change to higher K^+^ and lower Ca^2+^ concentrations. Under these conditions, APs were also induced in neurons from ASIC1a^–/–^ mice, in which a pH of ≤ 5.0 would be required to activate ASICs, indicating that ASIC activation was not required for the AP induction. Comparison between neurons of different ASIC genotypes indicated that the ASICs modulate the AP induction under such changed ionic conditions. Voltage-clamp measurements of the Na^+^ and K^+^ currents in cultured cortical neurons showed that the lowering of the pH inhibited Na^+^ and K^+^ currents. In contrast, the lowering of the Ca^2+^ together with the increase in the K^+^ concentration led to a hyperpolarizing shift of the activation voltage dependence of voltage-gated Na^+^ channels. We conclude that the ionic changes observed during high neuronal activity mediate a sustained AP induction caused by the potentiation of Na^+^ currents, a membrane depolarization due to the changed K^+^ reversal potential, the activation of ASICs, and possibly effects on other ion channels. Our study describes therefore conditions under which slow pH changes induce neuronal signaling by a mechanism involving ASICs.

## Introduction

The pH of body fluids is tightly controlled. The extracellular pH is normally close to 7.4. Rapid local pH fluctuations occur for example in synapses during neuronal activity (Chesler, 2003; Boscardin et al., 2016; Soto et al., 2018). Slower tissue acidification accompanies inflammation and ischemia (Cobbe and Poole-Wilson, 1980). Extracellular acidification induces action potentials (APs) in neurons. The transient receptor potential cation channel subfamily V member 1 (TRPV1) is an important pH sensor in the peripheral nervous system (PNS). It is activated by pH ≤ 6.5, with a pH of half-maximal activation, pH_50_, of ∼5.3 (Caterina et al., 1997; Tominaga et al., 1998). Acid-sensing ion channels (ASICs) are low pH-activated channels that are widely expressed in the peripheral and central nervous system (CNS) (Wemmie et al., 2013; Kellenberger and Schild, 2015). Several studies have shown that TRPV1 and ASICs are the pH sensors for acid-induced AP generation in the PNS (Deval et al., 2003; Blanchard and Kellenberger, 2011), while acid-induced AP generation in the CNS depends essentially on ASICs (Baron et al., 2002; Vukicevic and Kellenberger, 2004). In contrast to its effect on TRPV1 and ASICs, extracellular acidification inhibits many excitatory ion channels, such as glutamate receptors and voltage-gated Ca^2+^ and Na^+^ channels (reviewed in Boscardin et al., 2016).

The main ASIC subunits are ASIC1a, −1b, −2a, −2b, −3, and −4 (Wemmie et al., 2013; Kellenberger and Schild, 2015). In rodents, ASIC1a, −2a, −2b, and −4 are expressed in the CNS, while all ASIC subunits except ASIC4 are found in the PNS. The hetero- or homotrimeric assembly of ASIC subunits forms Na^+^-selective ion channels (Yang and Palmer, 2014; Lynagh et al., 2017) that differ in their H^+^ sensitivity [pH_50_ values between 6.6 (ASIC3) and 4.3 (ASIC2a)] and kinetics (Wemmie et al., 2013; Kellenberger and Schild, 2015; Schuhmacher et al., 2015; Rook et al., 2020, 2021). ASIC4 has so far not been shown to form functional channels and may regulate the expression of other ASICs (Akopian et al., 2000; Gründer et al., 2000; Lin et al., 2015). ASIC currents are transient because these channels enter a non-conducting, desensitized state after opening (Kellenberger and Schild, 2015; Rook et al., 2021). ASIC3 and some heterotrimeric ASICs produce in addition to the transient also a smaller sustained current. Animal studies identified roles of ASICs in synaptic plasticity and learning, fear-related behaviors, pain sensation, neuronal death after ischemic stroke and in neurodegenerative diseases (Wemmie et al., 2003; Xiong et al., 2004; Coryell et al., 2007; Friese et al., 2007; Pignataro et al., 2007; Deval et al., 2008, 2011; Kreple et al., 2014; Chiang et al., 2015; Sluka and Gregory, 2015; Lee and Chen, 2018; Chang et al., 2019). ASICs are potential drug targets of high interest. However, none of the available ASIC inhibitors is currently used in clinic (Baron and Lingueglia, 2015; Rash, 2017).

Traditionally, rapid solution changes are used to activate ASICs in studies in which ASIC currents are measured. However, some of the physiological and pathological processes in which ASICs are involved, are associated with slow and sustained pH changes, as e.g., inflammatory pain sensation or neurodegeneration after ischemic stroke. In a recent study we have investigated the activation of recombinant ASIC1a and of endogenous ASICs of cultured cortical neurons by pH ramps of defined speed (Alijevic et al., 2020). This analysis showed a strong decrease of the acid-induced current amplitude when the pH change was slowed, because a fraction of the channels desensitized without opening. If the pH ramp from 7.4 to 6.6 or 6.0 lasted 4–10 s, it induced longer bursts of action potentials (APs) in cultured cortical neurons than did faster pH ramps. However, with pH changes that took > 10 s to complete, APs were only very rarely induced. It seemed therefore that additional, currently unknown modulatory mechanisms are needed to allow an ASIC activation with such slow pH changes.

ASIC function is modulated by many compounds. Short peptides containing an Arg-Phe motif add a small sustained current to the transient ASIC1a current and shift its pH dependence of steady-state desensitization (the transition from the closed to the desensitized state) to more acidic values (Askwith et al., 2000; Sherwood and Askwith, 2009; Vick and Askwith, 2015; Bargeton et al., 2019; Borg et al., 2020) and have similar effects on ASIC3-containing channels. Molecules related to 2-guanidine-4-methylquinazoline induce a sustained activity in ASIC3 (Li et al., 2011). Reducing agents were shown to increase, while oxidizing reagents inhibit ASIC currents (Andrey et al., 2005; Chu et al., 2006; Cadiou et al., 2007; Cho and Askwith, 2007). ASIC function is also modulated by divalent cations (Babini et al., 2002; Chu et al., 2004). It is known that under some conditions that induce acidification, the concentrations of other ions are also changed. During high neuronal activity, seizures and ischemia, the extracellular concentrations of H^+^ and K^+^ increase in the CNS, while the Ca^2+^ concentration decreases (Heinemann and Louvel, 1983; Krishtal et al., 1987; Chesler, 2003; Brocard et al., 2013; Sibille et al., 2015; Larsen et al., 2016). These changes in ion concentration occur within seconds to minutes. During high neuronal activity, the extracellular K^+^ concentration reaches 6–10 mM, while the extracellular Ca^2+^ concentration, on the other hand, decreases to values as low as 0.8 mM (Heinemann et al., 1977; Brocard et al., 2013; Raimondo et al., 2015; Sibille et al., 2015; Larsen et al., 2016). In the present study we have investigated whether the above-described changes of K^+^ and Ca^2+^ concentrations may affect acid-induced AP generation. We show that such changes in K^+^ and Ca^2+^ concentration accompanied by a pH change to pH7.0 or 6.8 induced APs in cultured cortical neurons, even if the solution change was slow. The expressed ASIC subtype shaped the response; however, ASIC activity was not required for AP induction under these circumstances. Analysis of the modulation of channel function suggests that changed properties of voltage-gated Na^+^ channels, together with a depolarization induced by the increase in extracellular K^+^ concentration, as well as the activation of ASICs contribute to the AP induction under these ionic conditions.

## Materials and Methods

### Embryonic Mouse Cerebral Cortex Neuron Culture

All animal handling procedures were carried out according to the Swiss federal law on animal welfare, and were approved by the committee on animal experimentation of the Canton de Vaud (License VD1750). ASIC1a^–/–^ and ASIC2^–/–^ mice were provided by Dr. John Wemmie (University of Iowa). The cortex neuronal culture was done as previously described (Alijevic et al., 2020). Cerebral cortices of fetuses of day E14–15 from C57BL/6 genetic background of WT, ASIC1a^–/–^ or ASIC2^–/–^ pregnant mice were dissected. They were placed in ice-cold HBSS medium (Thermo Fisher Scientific) complemented with 5 mM HEPES, chopped into small pieces (∼1 mm) and incubated at 37°C for 15 min in HBSS medium containing 0.05% Trypsin-EDTA (Thermo Fisher Scientific). The cortical tissues were then washed three times in Neurobasal medium (Thermo Fisher Scientific) containing 10% fetal calf serum (FCS) and dissociated to single cells by gentle trituration using 1 mL blue tips (cut to 0.4 cm diameter) in the Neurobasal/FCS medium containing additionally 1 mg/mL DNase (Sigma-Aldrich: DN25), before centrifugation at 1,000 rpm for 5 min. The neurons were re-suspended in Neurobasal/FCS medium and seeded at 40,000 –200,000 cells/dish on 35-mm petri dishes containing three 15-mm diameter glass coverslips previously coated with poly-L-lysine. After 3 h the medium was replaced by Neurobasal Medium, Electro (Thermo Fisher Scientific:) containing B27 serum-free supplement, Electro (Thermo Fisher Scientific), GlutaMAX supplement (Thermo Fisher Scientific) and gentamicin (10 mg/ml final concentration; Thermo Fisher Scientific). Neuronal cultures were maintained in an incubator (37°C, 5% CO_2_ in humidified air) and every 2–3 days half of the medium was replaced with fresh plating medium. Patch-clamp experiments of cortical neurons were performed in a Warner Instruments RC-42LP measuring chamber after at least 14 days post-seeding, to account for sufficient expression of both ASIC1 and ASIC2 channels (Li et al., 2010).

### Electrophysiological Measurements

**FIGURE 1:**
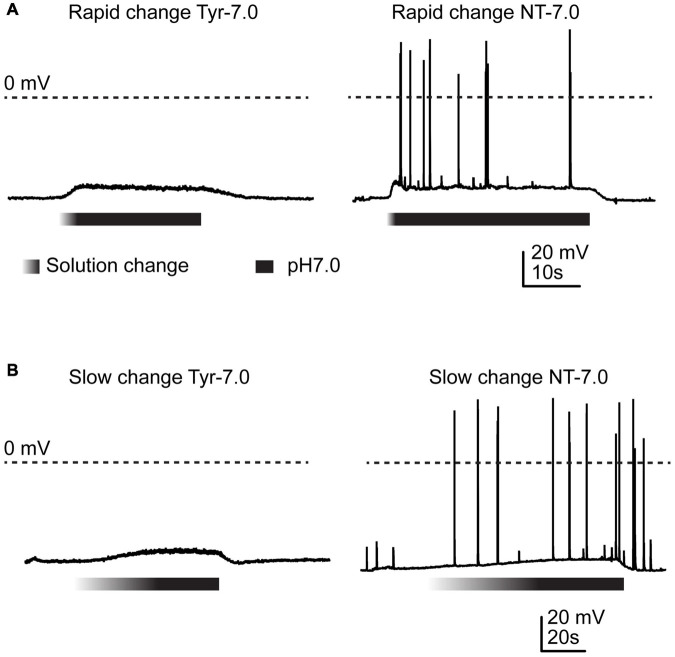
Action potential induction by changes in Ca^2+^ and K^+^ concentration. Current-clamp experiments with cortical neurons. Baseline current was injected to obtain a resting membrane potential close to –60 mV in the pH7.4 Tyrode (Tyr) solution. Solution change from Tyr solution at pH7.4 to either Tyr solution at pH7.0 or “neurotransmission” (NT) solution at pH7.0. The Ca^2+^ and K^+^ concentrations of the Tyr solution were 2 and 4 mM, respectively, and those of the NT solution 1 and 6 mM. The gradient part of the bar indicates the pH ramp, the black part the perfusion with pH7.0. **(A)** Rapid changes, **(B)** slow changes.

**FIGURE 2:**
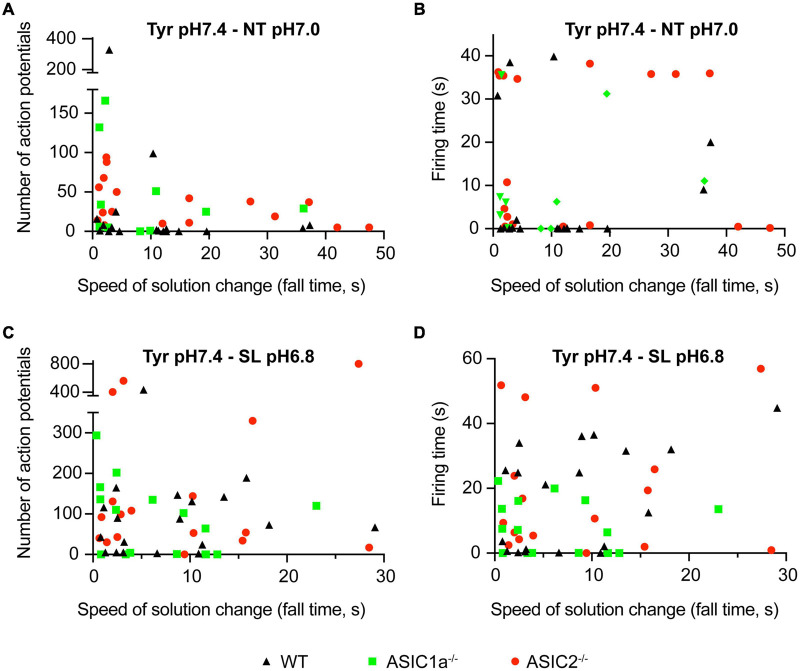
Dependence of AP number and firing time on the kinetics of solution change. Current-clamp experiments with cortical neurons. Baseline current was injected to obtain a resting membrane potential close to –60 mV in the pH7.4 Tyr solution. In these experiments the solution was changed from Tyrode solution at pH7.4 to either NT solution at pH7.0 or SL solution at pH6.8. In the Tyr solution, Ca^2+^ and K^+^ concentrations are 2 and 4 mM, respectively, while they are 1 and 6 mM in the NT and 0.8 and 8 mM in the SL solution. Number of APs **(A,C)** and the firing time (FT; **B,D**) are plotted as a function of ramp fall time of the solution change, as indicated; *n* = 12–21 per genotype and condition. The FT was determined as the time between the start of the first and the end of the last AP.

**FIGURE 3:**
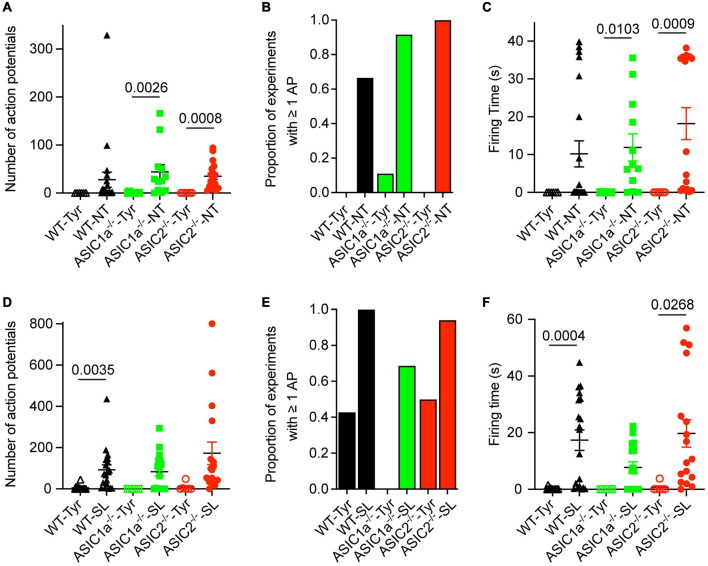
Analysis of AP number and firing time as a function of genotype and ion composition. The data were obtained from current-clamp experiments with cortical neurons as described in the legend to Figure 2. **(A,D)** Number of APs induced by solution changes from Tyr-pH7.4 to the solutions indicated, at pH7.0 **(A)** or pH6.8 **(D)**, in WT, ASIC1a^–/–^ and ASIC2^–/–^ cortical neurons (*n* = 6–21). **(B,E)** The proportion of experiments in which at least 1 AP was induced is indicated for the different conditions and genotypes, Tyr and NT solution at pH7.0 in **(B)** and Tyr and SL solution at pH6.8 in **(E)**, *n* = 6–21. **(C,F)** FT, determined as described in the legend to Figure 2, for the indicated experimental conditions [**(C)** pH7.0, **(F)** pH6.8], *n* = 6–21. For this analysis, the data solution changes with different kinetics were pooled, since the pH ramp kinetics did not influence the AP induction (Figure 2). The statistical significance of the differences between mean values was tested with the Kruskal-Wallis test, followed by Dunn’s multiple comparisons test.

**FIGURE 4:**
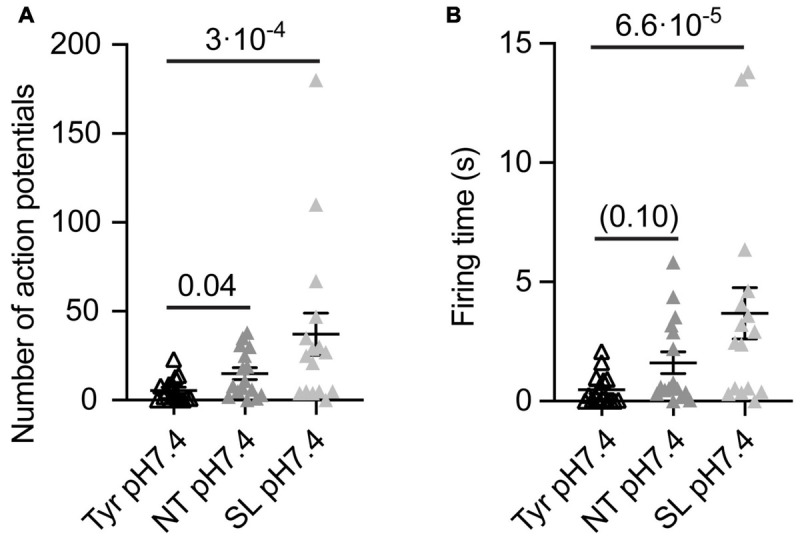
Analysis of AP number and firing time as a function of ion composition at pH7.4 from WT cortical neurons. The data were obtained from current-clamp experiments with cortical neurons as described in the legend to Figure 2. Note that in these experiments there was a signaling activity even at Tyr-pH7.4, probably due to a higher neuron density than in the experiments of Figures 1–3. The solutions were changed with a rapid solution change system from Tyr-pH7.4 to Tyr-pH7.4, NT-pH7.4 or SL-pH7.4 in each of the neurons tested to obtain a direct comparison between the three conditions of the number of APs **(A)** and the FT (**B**, *n* = 16). The statistical significance of the differences between mean values was tested with the Friedman test, followed by Dunn’s multiple comparisons test.

Electrophysiological measurements were carried out at room temperature (20–25°C) using the whole-cell configuration of the patch-clamp technique in voltage- and current-clamp mode with an EPC10 patch-clamp amplifier (HEKA Elektronik-Harvard Bioscience). Data were acquired with Patchmaster software and analysis of the currents and potentials was carried out with Fitmaster (HEKA Elektronik-Harvard Bioscience). The sampling interval and the low-pass filtering were set in voltage-clamp experiments to 0.02 ms and to 5 kHz, respectively, and in current-clamp experiments to 1 ms and 3 kHz (part of data of Figures 1–3) and 0.05 ms and 5 kHz (data for Figure 4 and most of the data of Figures 1–3). Recording pipettes were made of borosilicate glass (thin-wall capillaries, World Precision Instruments, United States) with a Narishige PC-10 puller and had resistances between 2 and 4 MΩ when filled with the pipette solution. In voltage-clamp experiments we set the series resistance compensation to 70–90% and the holding potential to −60 mV or as indicated.

Extracellular Tyrode solution contained (in mM): 140 NaCl, 4 KCl, 2 CaCl_2_, 1 MgCl_2_, 10 HEPES, 10 MES, 10 glucose; pH was adjusted to the desired value using NaOH/HCl. The two following solutions were designed to represent concentration changes of K^+^ and Ca^2+^ that are observed during high neuronal activity and during seizures (see main text). Extracellular “neurotransmission solution” (NT) contained (in mM): 137 NaCl, 6 KCl, 1 CaCl_2_, 1 MgCl_2_, 10 HEPES, 10 MES, 1 glucose. Extracellular “seizure-like” (SL) solution contained (in mM): 135 NaCl, 8 KCl, 0.8 CaCl_2_, 1 MgCl_2_, 10 MES, 10 HEPES, 10 glucose; Low Na^+^ extracellular solution contained (in mM): 30 NaCl, 114 TEA-Cl, 0.1 CdCl_2_, 2 BaCl_2_, 1 MgCl_2_, 10 MES, 10 HEPES, 10 glucose, 20 sucrose; Extracellular solution for K^+^ channel recording contained (in mM): 140 N-Methyl-D-Glucamine (NMDG), 4 KCl, 2 CaCl_2_, 1 MgCl_2_, 10 MES, 10 HEPES, 10 glucose, 30 sucrose. The pipette solution used for voltage-clamp experiments contained (in mM): 90 K gluconate, 10 NaCl, 10 KCl, 1 MgCl_2_, 60 HEPES, 10 EGTA; pH was adjusted to pH7.3 with KOH. The pipette solution for current-clamp experiments and for the experiments with voltage-gated currents contained (in mM): 90 K gluconate, 10 NaCl, 10 KCl, 3 MgCl_2_, 2 ATP-Na_2_, 0.3 GTP-Li^+^, 60 HEPES, 10 EGTA; pH was adjusted to pH7.3 with KOH. The intracellular solution used with low Na^+^ extracellular solution contained (in mM): 100 CsCl, 10 NaCl, 3 MgCl_2_, 10 EGTA, 2 ATP-Na_2_, 0.3 GTP-Li^+^, 60 HEPES; pH was adjusted to pH7.3 with CsOH. The pH of the solutions was controlled on the day of the experiment and adjusted if necessary. Rapid solution exchange was achieved using computer-controlled electrovalves (cF-8VS) and the MPRE8 perfusion head (Cell MicroControls, Norfolk, VA). pH ramps were generated using a pair of programmable pumps with 60 ml syringes (Aladdin syringe pump, World Precision Instruments, United States), whose output came together in a perfusion head. The hyperterminal program (Windows Operating System) was used for commanding the two syringe pumps. The total output rate of the two syringe pumps together was maintained constant at 100 ml/h. In current-clamp experiments, baseline current was injected to obtain a membrane potential close to −60 mV in presence of the pH7.4 Tyrode solution. This adjustment was made to start with similar resting membrane potential in all experiments. For the measurement of voltage-gated currents, the holding voltage was −90 mV, and currents were elicited by 100 ms steps of increasing amplitude to −90 to + 50 mV at 5 or 10 mV increments. The sweep interval was 4s. Leak current subtraction was done with the P/4 method.

### Extracellular pH Imaging

The speed of the solution change was determined for experiments in which pH ramps were applied. pH imaging experiments were performed using an inverted fluorescence microscope (Zeiss Axio Observer, AX10) with a 63x objective (Plan-Apochromat) and a CoolSnap HQ2 camera (Photometrics, United States). The time resolution of the imaging part was ∼ 440 ms. Images were recorded using the Metafluor software (v.7.3.3, Molecular Devices, Sunnyvale, United States). Briefly, cells were grown in Fluorodish cell culture dishes (World Precision Instruments, United States) or on 15-mm diameter glass coverslips (VWR, Germany) and incubated prior to the experiment in Tyrode solution containing 10 μM of 5(6)-FAM SE (5-(and-6)-Carboxyfluorescein, succinimidyl ester mixed isomers (BIOTIUM, United States), a ratiometric dye able to sense extracellular pH changes (excitation 460/488 nm, emission 520 nm) for 15 min in an incubator (37°C, 5% CO_2_). The amine-reactive succinimidyl ester form of the dye binds exclusively amine groups on cell surface proteins. The baseline of the 5(6)-FAM SE ratio measured during the perfusion of the tyrode solution showed a certain degree of photobleaching. We corrected for the photobleaching using Origin PRO software (OriginLab Corp., Northampton, United States). After baseline correction, the 90–10% fall time of the fluorescence signal was determined to classify the time course of the pH changes.

### Analysis and Statistics

The conductance-voltage curves of Na_*V*_s were fitted to a Boltzmann function: G(V) = G_*max*_/(1 + exp[(V−V_1/2_)/k], where G is the conductance, G_*max*_ the maximal conductance, V the voltage, V_1/2_ the voltage of half-maximal activation and k the slope factor. The conductance was calculated at each test voltage as G(V) = I/(V−V_*rev*_), where I is the current and V_*rev*_ the reversal potential obtained for each current-voltage (I–V) curve by linear interpolation or as indicated. The number of action potentials in current-clamp experiments was determined with FitMaster, measured over the same duration in pH ramps of different duration. The normality of the data distribution was determined by using the Shapiro-Wilk normally test. For normally distribution data we used student *t*-test for comparison between two groups or for paired comparisons and ANOVA followed by appropriate *post hoc* tests when more than two groups were involved. For not normally distribution data we used the Mann-Whitney test (for comparison between two groups) and the Kruskal-Wallis followed by Tukey *post hoc* test (or other appropriate tests, as indicated, for comparison between more than two groups). *P*-values of < 0.05 were considered significant. Data are presented as mean ± S.E.M.

## Results

### Changes to Solutions That Mimic the Composition Observed With High Neuronal Activity Induce Action Potentials

Several studies have shown that extracellular acidification induces a neuronal depolarization in neurons via the activation of ASICs, which leads to the generation of APs (Baron et al., 2002; Deval et al., 2003; Vukicevic and Kellenberger, 2004). We have recently shown that slowing of the pH change reduces the ASIC current amplitude and affects the ASIC-mediated AP induction in neurons (Alijevic et al., 2020). In this previous study we showed that acidification to pH6.6 or 6.0 in the presence of physiological ion concentrations induced APs in cultured mouse cortical neurons if the duration of the completion of the pH change was not longer than ∼10 s. As mentioned in the introduction, situations of high neuronal activity induce concentration changes of several ions, among them an increase in extracellular K^+^ and a decrease in extracellular Ca^2+^ concentration (Heinemann and Louvel, 1983; Frohlich et al., 2008; Brocard et al., 2013; Raimondo et al., 2015; Larsen et al., 2016). The difference in K^+^ concentration changes the equilibrium potential of K^+^ currents; a lowering of the extracellular Ca^2+^ concentration increases the pH sensitivity of ASICs (Babini et al., 2002), and strong reductions in Ca^2+^ concentrations were shown to induce a hyperpolarizing shift of the voltage dependence of activation of voltage-gated Na^+^ channels (Na_*V*_s) (Hille, 2001). Acidification has been shown to inhibit Na_*V*_ function (Hille, 2001). We reasoned that an acidification, if accompanied by changes in K^+^ and Ca^2+^ concentration, may induce even with slower pH ramps a long-lasting ASIC-mediated neuronal activity. To test this hypothesis, the Ca^2+^, H^+^ and K^+^ concentrations were simultaneously changed. When, in current-clamp experiments with physiological ion concentrations (“Tyrode” (Tyr) solution), the pH was rapidly changed from 7.4 to 7.0, generally no APs were induced (Figure 1A, left panel). Rodent cortical neurons express homotrimeric ASIC1a channels, heterotrimers composed of ASIC1a together with ASIC2a and/or ASIC2b, and ASIC2a homotrimers (Askwith et al., 2004; Wemmie et al., 2013; Kellenberger and Schild, 2015). The pH_50_ of these channels depends on the ASIC subunit combination. Homotrimeric ASIC1a has a pH_50_ of ∼6.4, while the pH_50_ of ASIC2a is ∼4.5; pH_50_ values of heteromeric ASIC1a/2 channels are in-between. We have previously measured a pH_50_ value of 6.3 in wild type (WT) mouse cortical neurons (Alijevic et al., 2020). This analysis showed that a pH change from 7.4 to 7.0 induced only very small ASIC currents that would probably not depolarize the membrane potential sufficiently to activate voltage-gated Na^+^ channels and induce action potentials, consistent with our observation in Figure 1A. If, however, the Tyrode solution at pH7.4 was changed to one at pH7.0 that mimics a situation of high neuronal activity, with 6 instead of 4 mM K^+^, and 1 instead of 2 mM Ca^2+^ (“neurotransmission” (NT) solution), action potentials were induced (Figure 1A, right panel). When the experiments described in Figure 1A were repeated with a slow pH change, the result was very similar (Figure 1B). In these experiments, the extracellular pH was gradually changed from 7.4 to 7.0 using two syringes for the perfusion, one at pH7.4 and one at 7.0 (Materials and methods). The solution at pH7.0 contained physiological K^+^ and Ca^2+^ concentrations for the experiment shown in the left panel, and 6 mM K^+^ and 1 mM Ca^2+^ for the experiment shown on the right.

Next it was determined whether the AP induction depended on the kinetics of the solution change from the Tyrode solution pH7.4 to the NT solution at pH7.0. To this end, experiments of the kind described in Figure 1 were carried out at different speeds of solution change. Figure 2A plots the number of induced APs as a function of the fall time (time to pass from 10 to 90%) of the solution change. This showed that for WT neurons (black triangles), there was no clear correlation between the kinetics of the solution change and the number of induced APs. As expected, the AP burst duration (“firing time,” FT, Figure 2B) did not show a dependence on the kinetics of the solution change either. Similarly to WT neurons, the number of induced APs and the FT did not depend on the kinetics of the solution change in ASIC1a^–/–^ and ASIC2^–/–^ neurons. In most measured neurons, the number of APs, and with it the FT, were relatively low. We had previously shown that acidification to 6.0 or 6.6 under physiological K^+^ and Ca^2+^ concentrations led to APs in WT and ASIC2^–/–^, but not in ASIC1a^–/–^ neurons (Alijevic et al., 2020). The absence of APs in ASIC1a^–/–^ neurons is due to the much lower pH sensitivity of ASICs formed by ASIC2 subunits. Changing to the NT solution at pH7.0 induced APs as expected in WT and ASIC2^–/–^ neurons. Surprisingly, the AP induction was also observed in ASIC1a^–/–^ neurons, suggesting that the ASIC activation is not required for NT solution-induced APs. As discussed above, the NT solution represents ionic conditions observed in high neuronal activity. During seizures, there occur even stronger changes in K^+^, Ca^2+^ and H^+^ concentrations (Raimondo et al., 2015; Boscardin et al., 2016). AP induction was in an additional set of experiments investigated for such a “seizure-like” (SL) condition (8 instead of 4 mM K^+^, 0.8 instead of 2 mM Ca^2+^ at pH6.8; Figures 2C,D). These experiments showed qualitatively similar results as observed with the NT solution, with a tendency of higher numbers of APs, however. Supplementary Figure 1A plots the depolarization, thus the difference between the basal membrane potential and the maximal membrane potential reached during the exposure to the test solution, as measured between the APs. A three-way ANOVA analysis confirmed as sources of variation the pH (*p* < 0.0001), the genotype (*p* = 0.0005) and the solution type (Tyr as compared to NT or SL; *p* = 0.0207). The figure illustrates the smaller depolarization induced by pH7.0 as compared to pH6.8 solutions. With changes to pH7.0, the depolarization appears higher with the NT as compared to the Tyr solution. The observation that acidification to pH6.8 in the Tyr solution induced a depolarization of 16 ± 2 mV (*n* = 6; Supplementary Figure 1A), suggests that these neurons may express other pH sensors than ASICs, or that the acidification may inhibit K^+^ channels (Boscardin et al., 2016). There was no clear correlation between the depolarization amplitude and the number of induced APs or the firing time (Supplementary Figures 1B,C). The overall conclusions of these experiments are that AP induction by NT and SL solutions 1) does not depend on the kinetics of the solution change and 2) does not require the activation of ASICs.

### Analysis of Action Potential Number and Firing Time of Pooled Data

Since the AP properties did not depend on the kinetics of solution change, data with different kinetics of solution change were pooled for the analysis and grouped according to the genotype and type of solution change. The FT values and numbers of APs were highly variable, as illustrated for the NT and SL conditions in Figure 2. Similarly to WT neurons, pH changes to the NT, but not the Tyrode solution of pH7.0, induced APs in ASIC1a^–/–^ and ASIC2^–/–^ neurons (Figure 3A). The increase in AP numbers in the NT compared to the Tyr solution was statistically significant in ASIC1a^–/–^ and ASIC2^–/–^ neurons. The plot of the proportion of experiments showing at least 1 AP (Figure 3B) further illustrates the effect of the changes in K^+^ and Ca^2+^ concentration. For changes to the Tyr solution at pH7.0, there were no APs in WT and ASIC2^–/–^ neurons; in one experiment with ASIC1a^–/–^ neurons, APs were induced. With the NT solution at pH7.0, at least one AP was observed in ∼65–90% of the experiments with WT and ASIC1a^–/–^ neurons, and in all experiments with ASIC2^–/–^ neurons. Due to the almost complete absence of APs with pH changes to pH7.0 in the Tyrode solution, the FT in this condition was 0s, while average FTs for experiments with APs in the NT condition were 10.2, 11.9 and 18.2 s for WT, ASIC1a^–/–^ and ASIC2^–/–^ neurons, respectively (Figure 3C). These values were significantly higher in the NT than the Tyr condition in ASIC1a^–/–^ and ASIC2^–/–^ neurons. Analogous experiments with the SL solution at pH6.8 showed higher numbers of APs compared to the Tyr solution at pH6.8 in WT neurons, and tendencies of higher numbers in ASIC1a^–/–^ and ASIC2^–/–^ neurons (*p* = 0.059 for ASIC1a^–/–^ and *p* = 0.0518 for ASIC2^–/–^ neurons; Figure 3D). In contrast to the experiments with acidification to pH7.0, the pH change to pH6.8 induced also with the Tyr solution ≥ 1 AP in ∼40% of the experiments with WT and ASIC2^–/–^ neurons. In ASIC1a^–/–^ neurons, no APs were induced in these conditions (Figure 3E). The change to the SL condition at pH6.8 induced APs in almost all WT and ASIC2^–/–^ neurons, and in 70% of ASIC1a^–/–^ neurons. The FT was < 1 s for the Tyr solution at pH6.8 in WT and ASIC2^–/–^ neurons, and 17.4, 7.7, and 19.7 s for WT, ASIC1a^–/–^ and ASIC2^–/–^ neurons, respectively, with the SL condition (Figure 3F); this increase was statistically significant for WT and ASIC2^–/–^ neurons. The AP numbers and FTs were not significantly different between the SL and the NT condition for a given genotype.

To evaluate the impact of the Ca^2+^ and K^+^ changes alone on the neuronal excitability, we measured the number of APs and FT induced by solution changes from Tyr-pH7.4 to Tyr-pH7.4, NT-pH7.4, or SL-pH7.4 solutions in WT cortical neurons. In this set of experiments, we observed a basal signaling activity even in the Tyr-pH7.4 solution, most likely due to higher neuronal density and consequently an increased number of interneuronal connections than in the previous cultures. In the absence of any pH changes, a higher number of APs, and a tendency to longer FTs was observed in NT as compared to the Tyr solution (Figures 4A,B). In SL solution at pH7.4, the number of APs and FT increased with regard to the Tyr solution (Figures 4A,B). This indicates that the increased signaling activity in NT and SL solutions did not require the acidification. Switching from the Tyr-pH7.4 to the NT-pH7.4 or the SL-pH7.4 solution induced in these experiments a sustained depolarization of 6.2 ± 0.4 and 12.7 ± 0.4 mV, respectively (*n* = 16).

Our experiments show that concomitant changes of H^+^, Ca^2+^, and K^+^ ion concentrations, as they occur during high neuronal activity or epileptic seizures, can induce long bursts of action potentials in cultured mouse cortical neurons, independently of the speed of solution change. In the two particular conditions that were studied here (corresponding to high neutrotransmission and seizure-like situations), the acidification was less important for the AP induction than was the change in Ca^2+^ and K^+^ ion concentrations. In the following we investigated which changes in ion channel function led to the observed increased excitability in NT and SL conditions.

### Lowering of the pH Induces Small Changes of Voltage Dependence and Current Amplitude of Na_*V*_s

**FIGURE 5:**
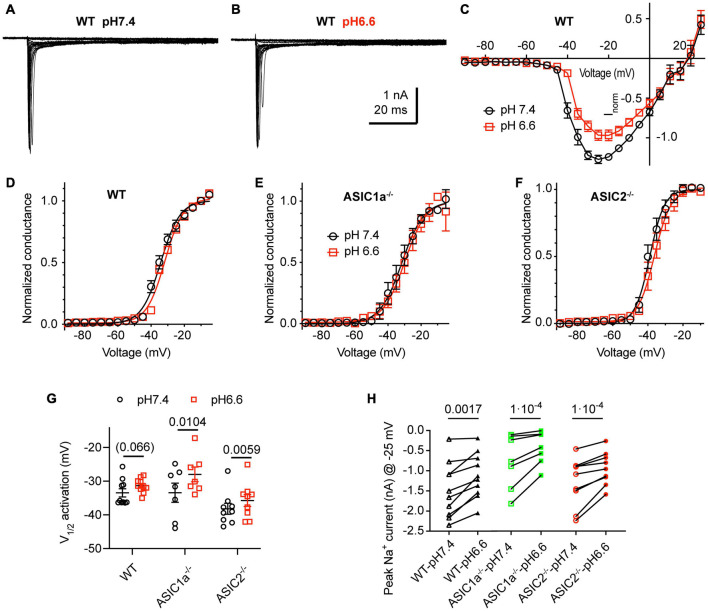
Acidification to pH6.6 induces a small inhibition of voltage-gated Na^+^ channels. Experiments were done in whole-cell voltage-clamp on cortical neurons. The voltage protocols are described under *Materials and methods*. The response to two different solutions was directly compared in the same cell. Only the pH was changed between the two conditions. **(A,B)** Representative current traces obtained at pH7.4 **(A)** and pH6.6 **(B)** from the same WT neuron **(C)** Na_*V*_ I-V curve at pH 7.4 and 6.6. Current amplitudes were normalized in each cell to the amplitude measured at pH7.4 at –10 mV, *n* = 10. **(D–F)** Na_*V*_ conductance-voltage curve at pH 7.4 and 6.6. The conductance was normalized to the conductance at –10 mV. **(D)** Neurons from WT mice. The V_1/2_, determined from a Boltzmann fit, was –33.5 ± 1.2 mV (pH7.4) and –31.3 ± 0.7 mV (pH6.6, *p* = 0.066, *n* = 10). **(E)** Neurons from ASIC1a^–/–^ mice. The V_1/2_ values were –33.4 ± 2.8 mV (pH7.4) and –28.0 ± 2.2 mV (pH6.6, *p* = 0.0104, *n* = 7). **(F)** Neurons from ASIC2^–/–^ mice. The V_1/2_ values were –38.2 ± 1.6 mV (pH7.4), –35.7 ± 1.8 mV (pH6.6, *p* = 0.0059, *n* = 9). **(G)** Plot of the V_1/2_ values obtained in experiments described in **(D–F)**. **(H)** Na_*V*_ current amplitude at –25 mV, measured from WT, ASIC1a^–/–^ and ASIC2^–/–^ cortical neurons, *n* = 7–10. Data of Figure 5 were obtained in an extracellular solution with decreased Na^+^ concentration to improve the clamp control (Materials and methods). *P*-values derived from paired *t*-tests are indicated in **(G,H)**.

We analyzed first how the pH changes used to activate ASICs and induce APs affect the voltage-gated Na^+^ and K^+^ channels. Figures 5A–C shows Na_*V*_ I–V curves obtained from WT neurons in a solution with physiological K^+^ and Ca^2+^ concentrations at pH7.4 and 6.6. The pH6.6, which is 0.2 units below the value of the more extreme condition investigated in current-clamp experiments, was chosen to make sure not to miss any effect of the pH. For these experiments, the Na^+^ concentration had been lowered to 30 mM (section “Materials and Methods”) to allow a better voltage-clamp of the currents. Changing the extracellular pH from pH7.4 to 6.6 showed in direct comparison a tendency of a depolarizing shift with acidification in WT neurons (Figures 5C,D; V_1/2_, pH7.4: −33.5 ± 1.2 mV and pH6.6, −31.3 ± 0.7 mV, *n* = 10, *p* = 0.066). A similar, statistically significant shift was observed in cortical neurons of ASIC1a^–/–^ and ASIC2^–/–^ mice (Figures 5E–G). The peak amplitude of Na_*V*_ currents, measured at −25 mV, was decreased by acidification to pH6.6 in WT, ASIC1a^–/–^ and ASIC2^–/–^ cortical neurons (Figure 5H). This inhibition amounted to 24 ± 4% in WT (*n* = 10), 37 ± 4% in ASIC1a^–/–^ (*n* = 7) and 24 ± 3% in ASIC2^–/–^ neurons (*n* = 9).

### Small Effects of pH on K^+^ Currents

**FIGURE 6:**
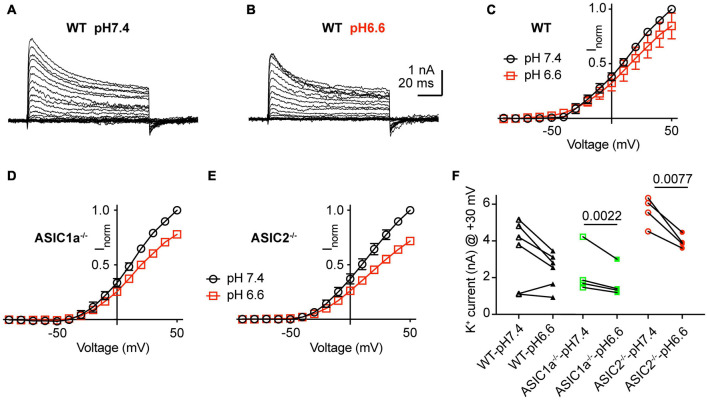
Inhibition of K^+^ currents by acidification to pH6.6. Experiments were done in whole-cell voltage-clamp on cortical neurons. The response to two different solutions was directly compared in the same cell. Only the pH was changed between the two conditions. **(A,B)** Representative current traces obtained at pH7.4 **(A)** and pH6.6 **(B)** from the same neuron. **(C–E)** I–V curve of K^+^ currents, measured at pH7.4 and pH6.6. Current amplitudes were normalized in each cell to the amplitude measured at pH7.4 at + 50 mV. **(C)** Neurons from WT mice, *n* = 6, **(D)** ASIC1a^–/–^ mice, *n* = 4 and **(E)** ASIC2^–/–^ mice, *n* = 4. **(F)** Peak K^+^ current amplitudes measured at + 30 mV, at pH7.4 and 6.6, from WT, ASIC1a^–/–^ and ASIC2^–/–^ cortical neurons, *n* = 4–6. *P*-values obtained with paired *t*-tests are indicated in **(F)** if they were < 0.05.

Acidification from pH7.4 to 6.6 led to an apparent decrease in K^+^ current amplitudes as illustrated in representative current traces and the I-V curves (Figures 6A–E). Since the reversal potential could not be measured reliably due to the small inward currents, conductance-voltage (G-V) curves were constructed based on a reversal potential of −83 mV, calculated from the nominal K^+^ concentrations in the measuring solutions (Supplementary Figure 2). The G-V curves obtained at pH7.4 and 6.6 from the same neurons indicated that the acidification does not affect the voltage dependence. Comparison of the current amplitudes at + 30 mV showed decreased values at pH6.6 (Figure 6F). This difference was significant in ASIC1a^–/–^ and ASIC2^–/–^, but not WT neurons. Inspection of the I-V curves at pH7.4 and 6.6 shows that the ratio of the current amplitude between the two conditions is the same over the voltage range of −20 to + 50 mV, and thus the difference between the amplitudes measured at the two conditions increases with positive voltage.

### Changes of Ca^2+^ and K^+^ Concentrations Affect Na_*V*_ Currents

**FIGURE 7:**
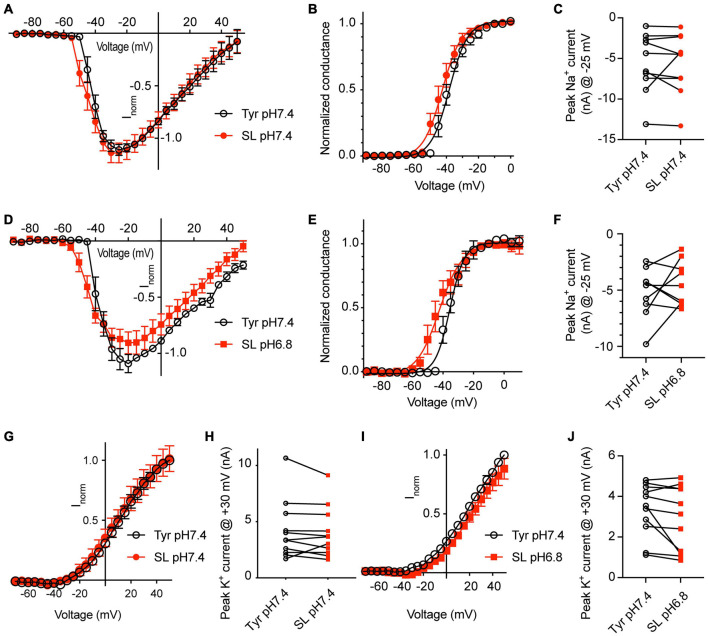
Solutions mimicking seizure-like conditions change the voltage dependence of voltage-gated Na^+^ channels. Experiments were done in whole-cell voltage-clamp on cortical neurons from WT mice. The response to two different solutions was directly compared in the same cell. **(A–C)** Between the two conditions, only the Ca^2+^ and K^+^ concentration, but not the pH was changed. **(A)** Na_*V*_ I-V curve in Tyr-pH7.4 or SL-pH7.4 solution. Current amplitudes were normalized in each cell to the amplitude measured in the Tyr solution at –10 mV, *n* = 10. **(B)** Na_*V*_ conductance-voltage curve in Tyr-pH7.4 or SL-pH7.4 solution. The conductance was normalized for each condition to the conductance determined at –10 mV. The V_1/2_ values were –39.0 ± 1.6 mV (Tyr) and –42.3 ± 2.1 mV (SL, *p* = 0.041, *n* = 10, paired *t*-test). **(C)** Na_*V*_ peak current amplitudes at –25 mV, measured in Tyr and SL solution at pH7.4 (*n* = 10). **(D–F)** Comparison of Na_*V*_ properties between Tyr-pH7.4 and SL-pH6.8 conditions in paired experiments. **(D)** Na_*V*_ I-V curve in Tyr-pH7.4 or SL-pH6.8 solution from paired experiments. Current amplitudes were normalized in each cell to the amplitude measured in the Tyr solution at –10 mV, *n* = 10. **(E)** Na_*V*_ conductance-voltage curve in Tyr-pH7.4 or SL-pH6.8 solution. The conductance was normalized for each condition to the conductance determined at –10 mV. The V_1/2_ values were –34.2 ± 2.1 mV (Tyr-pH7.4) and –41.0 ± 3.0 mV (SL-pH6.8). **(F)** Na_*V*_ peak current amplitudes at –25 mV, measured in Tyr-pH7.4 and SL-pH6.8 solution (*n* = 10). **(G)** I–V curve of K^+^ currents, measured in Tyr-pH7.4 and SL-pH7.4 solution, *n* = 11. **(H)** K^+^ peak amplitude, measured at + 30 mV, in Tyr-pH7.4 and SL-pH7.4 solution, *n* = 11. **(I)** I–V curve of K^+^ currents, measured in Tyr-pH7.4 and SL-pH6.8 solution, *n* = 11. **(J)** K^+^ peak amplitude at + 30 mV, measured in Tyr-pH7.4 and SL-pH6.8 solution, *n* = 11. The current amplitudes compared in **(C,F,H,J)** were not different between the two conditions tested. **(G,I)** In each cell, the current amplitudes measured were normalized to the amplitude measured in the Tyr-pH7.4 condition at +50 mV.

To determine the effect of changes in Ca^2+^ and K^+^ concentrations on Na_*V*_ currents, a first series of experiments was carried out at pH7.4, either in physiological extracellular Tyrode solution, or in a solution with 0.8 mM Ca^2+^ and 8 mM K^+^, mimicking thus in this regard a seizure-like condition (SL solution). These experiments were carried out with WT neurons. The change to the SL solution led to a hyperpolarizing shift of the voltage dependence of activation (Figures 7A,B; V_1/2_, Tyr-pH7.4, −39.0 ± 1.6 mV; SL-pH7.4, −42.3 ± 2.1 mV, *p* = 0.041, *n* = 10). The SL solution did not significantly change the Na_*V*_ peak current amplitude measured at −25 mV (Figure 7C). To test the consequences of the combined effect of Ca^2+^ and K^+^ concentration changes and a lowering of the pH, Na_*V*_ currents were measured in a second set of paired experiments in Tyr-pH7.4 and SL-pH6.8 solutions (Figures 7D–F). The voltage dependence of activation was shifted negatively in the SL-pH6.8 solution (Figure 7E, V_1/2_, Tyr-pH7.4, −34.2 ± 2.1 mV; SL-pH6.8, −41.0 ± 2.9 mV, *p* = 0.0083, *n* = 10). Although the I-V curves suggests slightly smaller current amplitudes in the SL-pH6.8 condition, the Na_*V*_ current amplitudes at −25 mV were not significantly different between the two conditions (Figure 7F). The hyperpolarizing shift of the activation voltage dependence in the SL-pH6.8 condition is expected to contribute to an increased excitability of the cortical neurons. Thus, the lowering of the Ca^2+^ together with the increasing of the K^+^ concentration has an opposite effect on the Na_*V*_ function compared to the acidification alone. Our analysis shows that the hyperpolarizing shift of the voltage dependence predominates when these changes in Ca^2+^ and K^+^ concentrations are combined with an acidification to pH6.8.

For the K^+^ currents, changing at pH7.4 from normal Tyrode to SL solution did not affect the voltage dependence, nor the current amplitudes (Figures 7G,H). If this change was accompanied by an acidification to pH6.8, apparently smaller current amplitudes were observed in the SL-pH6.8 condition (Figure 7I) that were however not significantly different from the current amplitudes measured in the Tyr-pH7.4 solution in paired experiments (Figure 7J).

## Discussion

Extracellular acidification induces neuronal activity via the activation of pH-sensing ion channels. ASICs are important pH sensors of the nervous system. It was documented in several studies that they contribute to the sensation of inflammatory pain and to neurodegeneration after ischemic stroke, two processes associated with slowly developing pH changes. In a recent study we showed that pH changes from pH7.4 to 6.6 or 6.0 that take more than 10 s to complete are unlikely to induce APs in neurons of the CNS (Alijevic et al., 2020). Since conditions of high neuronal activity and ischemia are associated with concentration changes of other ions besides protons, we tested here whether concentration changes of Ca^2+^ and K^+^ ions modulated acid-induced AP generation. We observed that small acidifications combined with lowering of the Ca^2+^ and increasing of the K^+^ concentration induced APs efficiently, independently of the speed of solution change. This AP induction did not require ASICs, was however modulated by ASICs. Investigation of the effects of these concentration changes on voltage-gated Na^+^ and K^+^ currents showed an inhibition of Na^+^ and K^+^ channels by the lowering of the pH, and a hyperpolarization of the activation voltage dependence of Na_*V*_s if the acidification was combined with a lowering of the Ca^2+^ and an increase of the K^+^ concentration.

### Effects of the Acid-Sensing Ion Channel Genotype

The experiments in which the speed of the change to the NT or SL solution was varied, indicated that the number of induced APs and the FT did not depend on the kinetics of the changes in ion concentrations, in opposition to the limitation observed in experiments with solutions of physiological ion concentrations (Alijevic et al., 2020), in which the AP induction required ASIC activation.

The ASIC subunits that can contribute to functional ASICs in CNS neurons are ASIC1a, ASIC2a, and ASIC2b. ASIC2b cannot form pH-activated homotrimeric channels but participates in the formation of heteromers (Lingueglia et al., 1997). Homotrimeric ASIC1a has a higher H^+^ sensitivity than ASIC2a, as discussed above. In CNS neurons of ASIC1a^–/–^ mice, lower pH values than pH5.0 are required to induce ASIC currents (Askwith et al., 2004). CNS neurons of ASIC2^–/–^ mice have ASICs that are more sensitive to pH than is found in WT neurons, because functional ASICs in this background are essentially ASIC1a homotrimers. The pH_50_ of ASIC activation in cortical neurons of WT mice was ∼6.3, while this value was ∼6.7 in ASIC2^–/–^, and < 4.3 in ASIC1a^–/–^ neurons (Alijevic et al., 2020). These pH_50_ values in the ASIC knockout neurons are close to the reported pH_50_ values of recombinant ASIC1a and ASIC2a, respectively, strongly suggesting that there is no compensatory upregulation of other ASIC subunits in the knockout animals. Changes to the Tyr solution induced APs in ≤ 10% of the experiments, if the final pH was 7.0. In contrast, a change to the Tyr solution at pH6.8 induced APs in ∼40% of the experiments in WT and ASIC2^–/–^ neurons, consistent with previous observations that acidification to pH6.8 induces small ASIC currents. In ASIC1a^–/–^ neurons, APs were not induced with the pH6.8 Tyr solution, because of the reduced pH sensitivity of the ASIC1a-less channels.

The NT and SL solutions at pH7.0 and 6.8, respectively, induced APs also in neurons from ASIC1a^–/–^ mice, suggesting that the activation of ASICs was not required for AP induction. The ASIC genotype of the neurons influenced the AP induction in the Tyr-pH6.8 condition. We did, however, not observe any significant differences between the ASIC genotypes regarding the AP induction with the NT and SL solutions. The AP generation in neurons depends on many factors, resulting in a high neuron-to-neuron variability, which may have precluded the detection of a contribution of ASICs to AP induction in the other test conditions. It is expected that with smaller concentration changes of Ca^2+^ and K^+^ than tested here, the activation of ASICs by the acidification would contribute more significantly to the AP generation.

In a separate set of experiments in WT neurons, we have directly compared AP induction by the Ca^2+^ and K^+^ changes alone, without concomitant acidification. The comparison of the three solutions in the same neurons indicates that the lowering of the Ca^2+^ together with the increase of the K^+^ concentration increased the signaling activity independently of an acidification.

### Changes in Ion Concentrations Observed During High Neuronal Activity and Seizures

Variations in interstitial ion concentrations in the brain occur under many physiological conditions (Ding et al., 2016). Higher concentration changes were observed under pathological conditions. The basal extracellular K^+^ concentration in brain was measured as 3–4 mM. During high neuronal activity, such as seizures, this concentration increased up to 10, maximally 12 mM (Heinemann and Louvel, 1983; Frohlich et al., 2008; Brocard et al., 2013; Raimondo et al., 2015; Larsen et al., 2016). Basal Ca^2+^ concentrations in the brain were between 1 and 2 mM, in many studies close to 1.2 mM. During high neuronal activity, this concentration decreased to ∼0.8 mM (Heinemann and Louvel, 1983; Brocard et al., 2013) or even lower concentrations (Raimondo et al., 2015). Following an ischemic stroke, even more dramatic changes in Ca^2+^ and K^+^ concentrations were observed (Kristian and Siesjo, 1998; Mori et al., 2002). An increase of the extracellular K^+^ concentration depolarizes the neuronal membrane potential, decreases input resistance, and can activate bursting in some neuron types (reviewed in Frohlich et al., 2008). On the level of the neuronal circuitry, increases in K^+^ concentration reduce synaptic inhibition, because the K^+^-Cl^–^-cotransporter KCC2 will operate with a reduced electrochemical driving force, and extrude less Cl^–^ (Frohlich et al., 2008). Consequently, the GABAergic transmission might show excitatory effects as long as the increased extracellular K^+^ concentration is able to maintain a reduced KCC2 activity. The resting pH value of the interstitial fluid of the brain is generally 0.1–0.2 pH units more acidic than the pH of the blood (Mutch and Hansen, 1984; Syková and Svoboda, 1990). Repetitive electrical stimulation induced in several studies first a short alkalinization of 0.01–0.2 pH units, which was followed by a slower developing, long-lasting acidic shift of 0.1–0.25 pH units (Chesler, 1990, 2003; Makani and Chesler, 2010). The acidification occurring in synapses was estimated to 0.2–0.6 pH units (DeVries, 2001; Vessey et al., 2005). A study with rats showed a brain tissue acidification induced by seizures of ∼0.36 pH units in normoxic, and of 0.51 pH units in moderately hypoxic animals (Siesjö et al., 1985).

One of the consequences of the extracellular acidification is dampening the excitatory neurotransmission, while the concomitant reduction of external calcium and rise of potassium ions appear to have a counterbalancing effect for restoring the excitatory tone. The changes in Na_*V*_ function that we observed with the SL solution are consistent with earlier studies that investigated the dependence of Na_*V*_ function on the Ca^2+^ concentration. Reducing the extracellular Ca^2+^ concentration shifts the conductance-voltage relationship of Na_*V*_s and other voltage-gated channels to more hyperpolarized values (Campbell and Hille, 1976; Hille, 2001). In frog skeletal muscle, lowering the extracellular Ca^2+^ concentration from 2 to 0.5 mM shifted the conductance-voltage curve by −8 mV (Campbell and Hille, 1976). This effect may be due to reduced surface charge screening (Hille, 2001). The extracellular Ca^2+^ concentration co-defines also the pH dependence of ASICs. There appears to be a competition between Ca^2+^ and H^+^. Therefore, lowering of the Ca^2+^ concentration induces an alkaline shift of the pH dependence of activation and steady-state desensitization (Babini et al., 2002). Ca^2+^ concentration changes between 2 and 0.5 mM appear to induce rather small shifts in ASIC1a pH dependence (Babini et al., 2002). Millimolar Ca^2+^ concentrations are known to lead to a weak pore block of several ion channels, as Na_*V*_s and ASICs among others. For ASIC1a, the IC_50_ for inhibition is ∼5 mM (Paukert et al., 2004).

Extracellular protons have been shown to induce a pore block of Na_*V*_ currents. A midpoint of inhibition between pH4.6 and 5.4 has been reported for amphibian Na_*V*_s (reviewed in Hille, 2001). Extracellular pH is known to affect also the voltage dependence of Na_*V*_s. In amphibian nerve fibers, lowering the extracellular pH to values < 5.5 induced a depolarizing shift of the voltage dependence of activation (Hille, 1968). Acidification to pH6.0 induced a ∼30% reduction of the current amplitude and did not affect the voltage dependence of recombinantly expressed rat Na_*V*_1.2 (Vilin et al., 2012). An earlier study using rat CA1 neurons showed that a change from pH7.4 to 6.4 induced a small depolarizing shift in the voltage dependence of activation and did not affect steady-state inactivation and current kinetics (Tombaugh and Somjen, 1996). Our observations are consistent with these earlier findings obtained in different organisms and cell systems at more acidic pH ranges.

Another prominent ion channel type whose activity depends on the extracellular Ca^2+^ concentration is the calcium homeostasis modulator 1 (CALHM1), which is permeable to monovalent cations and to Ca^2+^, and is expressed in mouse cortical neurons. Its activity depends on the membrane voltage and the extracellular Ca^2+^ concentration (Ma et al., 2012, 2016). The IC_50_ for Ca^2+^ inhibition of CALHM1 was estimated as ∼0.2 mM at −60 mV (Ma et al., 2012), suggesting that CALHM1 would only be partially potentiated by the Ca^2+^ concentration changes investigated in our study.

### Changing to Neurotransmission or Seizure-Like Solutions Increases Neuronal Excitability

The increased AP induction with NT and SL solutions is due to the effects of the H^+^, K^+^, and Ca^2+^ concentrations on different targets. Our voltage-clamp analysis of Na^+^ and K^+^ channels provided the following information. Lowering of the pH to 6.6 induced in our experimental conditions a decrease of the Na_*V*_ current amplitude, in accordance with previous studies, as well as a small depolarizing shift of the activation voltage dependence. It decreased in addition the amplitude of K^+^ currents. If the pH was kept at 7.4, but the K^+^ and Ca^2+^ concentrations were changed to the SL conditions, thus from 4 to 8 mM for K^+^, and from 2 to 0.8 mM for Ca^2+^, the Na_*V*_ current amplitude was not affected, and the activation voltage dependence was shifted to more hyperpolarized values. If these changes in K^+^ and Ca^2+^ concentrations were combined with an acidification to pH6.8, which corresponds to the SL-pH6.8 condition tested in the current-clamp experiments of Figures 2, 3, the effect of the K^+^ and Ca^2+^ concentration changes was predominant over the effect of the acidification, resulting thus in a hyperpolarizing shift, which is expected to increase the neuronal excitability, consistent with the observed effects on AP induction. The K^+^ current amplitudes were not affected by the change to the SL conditions, although this solution change induced a theoretical shift of + 18 mV of the reversal potential, and with this a reduced driving force.

In situations in which the H^+^, K^+^, and Ca^2+^ concentrations change, the effect of acidification on ASICs can induce APs if acidification is fast, or prevent ASIC-mediated APs, if the acidification is slow, because of desensitization of ASICs (Alijevic et al., 2020), while the effects of K^+^ and Ca^2+^ concentration changes on neuronal excitability do not depend on the speed of the solution change. Such changes in K^+^ and Ca^2+^ concentration will allow a contribution of ASICs to neuronal signaling under pH conditions in which ASICs are normally not or not sufficiently activated. The different dependence of H^+^-induced ASIC activity and of K^+^ and Ca^2+^ concentration-dependent changes in neuronal signaling on the kinetics of the concentration changes add an additional level of complexity to the regulation of AP signaling.

In conclusion, the efficient induction of APs by the NT and SL solutions in our experiments is therefore due in part to changed properties of Na_*V*_s, for which the positive modulation by the decrease of the Ca^2+^ concentration is stronger than the negative modulation by the increase in H^+^ concentration. The increased K^+^ concentration led to a depolarization of the membrane potential. On ASICs, the lowering of the Ca^2+^ concentration shifted the pH-response curve to more alkaline values, while the acidification led to a partial activation, at least in the SL condition. It is highly probable that these ion concentration changes also affect other channels or transporters that may further contribute to the changed excitability. Our study describes conditions under which an acidification, independently of the speed of the solution change, induces APs. Although the ASICs were not required for the AP induction, they contributed to it in some of the tested conditions.

## Data Availability Statement

The original contributions presented in the study are included in the article/Supplementary Material, further inquiries can be directed to the corresponding author/s.

## Ethics Statement

The animal study was reviewed and approved by the committee on animal experimentation of the Canton de Vaud, Service vétérinaire.

## Author Contributions

OA and SK designed the project and wrote the manuscript. ZP and OA carried out the experiments. All authors approved the final manuscript.

## Conflict of Interest

The authors declare that the research was conducted in the absence of any commercial or financial relationships that could be construed as a potential conflict of interest. The reviewer, LR, declared a shared committee with one of the authors, SK, at the time of review.

## Publisher’s Note

All claims expressed in this article are solely those of the authors and do not necessarily represent those of their affiliated organizations, or those of the publisher, the editors and the reviewers. Any product that may be evaluated in this article, or claim that may be made by its manufacturer, is not guaranteed or endorsed by the publisher.

## Abbreviations

AP: action potential
ASIC: acid-sensing ion channel
FCS: fetal calf serum
FT: firing time
G-V curve: conductance-voltage curve
I-V curve: current-voltage curve
Na_*V*_: voltage-gated Na^+^ channel
NMDG: N-Methyl-D-Glucamine
NT: neurotransmission
pH_50_: pH of half-maximal activation
SL: seizure-like
TRPV1: transient receptor potential cation channel subfamily V member 1
Tyr: tyrode
V_1/2_: voltage of half-maximal activation
WT: wild-type.
