# Supplementary material for: Changes in H+, K+, and Ca2+ Concentrations, as Observed in Seizures, Induce Action Potential Signaling in Cortical Neurons by a Mechanism That Depends Partially on Acid-Sensing Ion Channels
Source: Front Cell Neurosci. 2021 Oct 15;15:732869. doi: 10.3389/fncel.2021.732869 (PMC8553998; doi:10.3389/fncel.2021.732869)
Supplement: Supplementary file 2 [file Data_Sheet_2.pdf]

Supplementary Material

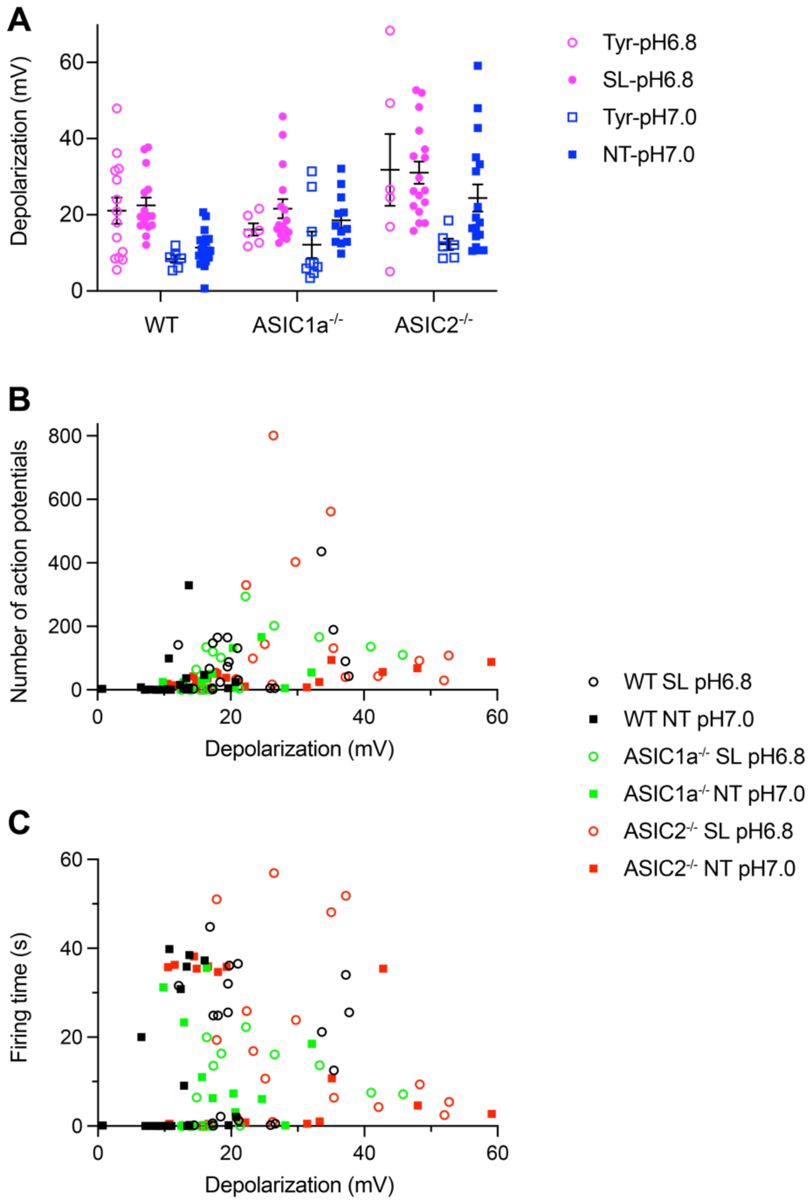

Supplementary Figure S1. Depolarization induced by pH, Ca^{2+} and K^{+} changes. The data were obtained from current-clamp experiments with cortical neurons as described in the legend to Figure 2. (**A**) The difference between the basal membrane potential and the maximal membrane potential reached during the solution change to the test solution, as measured between the action potentials, is plotted as a function of the genotype and ion concentration (n=6-21). Dependence of AP number (**B**) and firing time (in s; **C**) on the depolarization changes induced by the indicated solution change. A three-way ANOVA analysis identified as sources of variation the pH ($p<0.0001$), the genotype ($p=0.0005$) and the solution type (Tyr as compared to NT or SL solutions; $p=0.0207$).

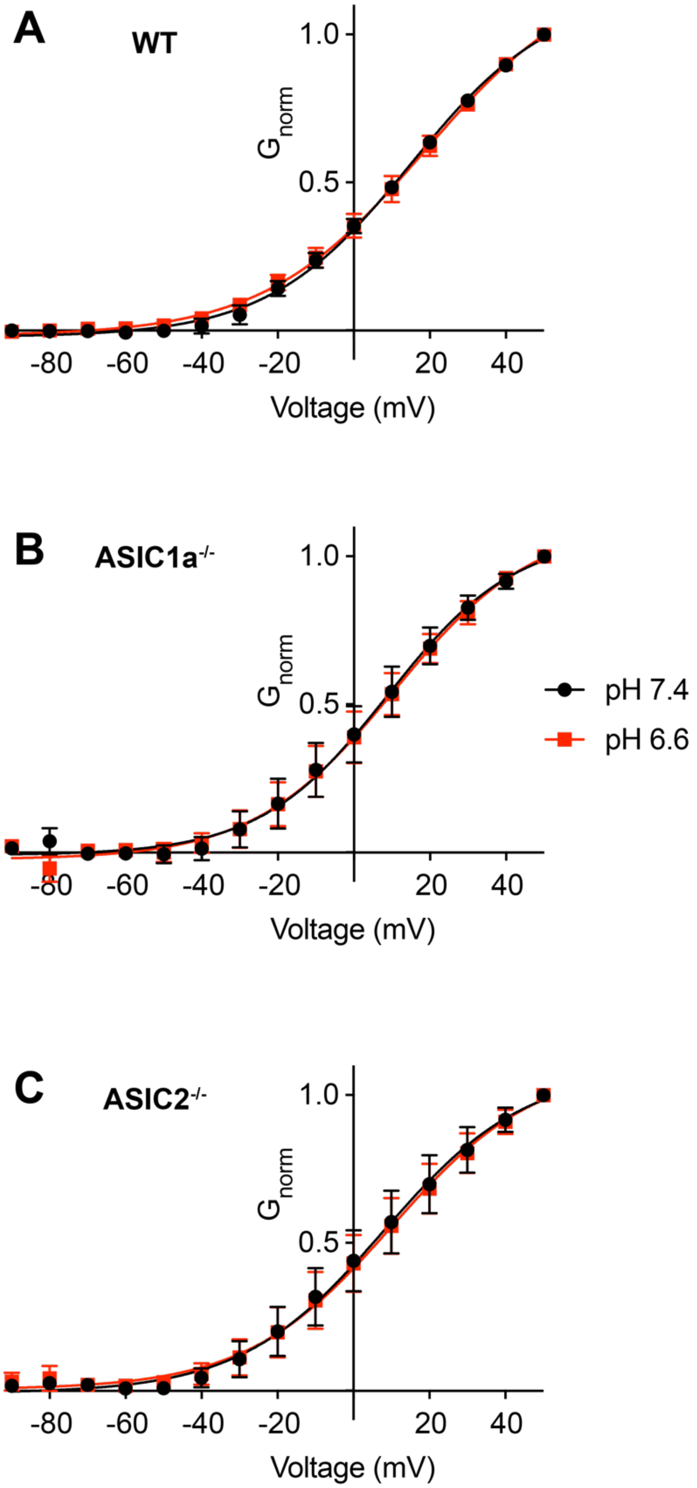

Supplementary Figure S2. K_V conductance-voltage curve at pH 7.4 and pH 6.6 in experiments of Figure 6. The conductance, calculated based on the theoretical reversal potential of -83mV, was normalized for each condition to the conductance determined at +50 mV, measured from WT (**A**), ASIC1a$^{-/-}$ (**B**) and ASIC2$^{-/-}$ (**C**) cortical neurons (n=4-5).
